# Supplementary material for: Genomic Surveillance of Salmonella from the Comunitat Valenciana (Spain)
Source: Antibiotics (Basel). 2023 May 9;12(5):883. doi: 10.3390/antibiotics12050883 (PMC10215213; doi:10.3390/antibiotics12050883)
Supplement: Supplementary file 1 [file antibiotics-12-00883-s001.zip › antibiotics-2362614-supplementary.pdf]

Supplementary Material

Table S1. Sampling information (product, location, and year) of the *Salmonella enterica* isolates included in this study.

Isolate	Product	Location	Year
Se_V_1	Pork	Benaguacil	2016
Se_V_2	Pork	Picassent	2017
Se_V_3	Turtle aquarium	Castellón	2012
Se_V_4	Pork	Valencia	2016
Se_V_5	Pork	Valencia	2016
Se_V_6	Pork	Valencia	2015
Se_V_7	Poultry	FoNA Carrós	2016
Se_V_8	Poultry	FoNA Carrós	2016
Se_V_9	Chicken	Denia	2013
Se_V_10	Chicken	Denia	2013
Se_V_11	Chicken	Elche	2016
Se_V_12	Chicken	Crevillent	2016
Se_V_13	Pork	Burriana	2016
Se_V_14	Chicken	Algemesí	2017
Se_V_15	Pork	Betxí	2017
Se_V_16	Chicken	Algemesí	2017
Se_V_17	Pork	Valencia	2017
Se_V_18	Pork	Valencia	2017
Se_V_19	Pasta salad	Benidorm	2017
Se_V_20	Chicken	Torrent	2017
Se_V_21	Pork	Borriana	2017

Se_V_22	Sausage	Pinoso	2016
Se_V_23	Sausage	Pinoso	2016
Se_V_24	Pork	Valencia	2016
Se_V_25	Pork	Xeresa	2016
Se_V_26	Pork	Sueca	2016
Se_V_27	Pork	Sueca	2016
Se_V_28	Pork	Valencia	2016
Se_V_29	Pork	Oliva	2016
Se_V_30	Pork	Picanya	2016
Se_V_31	Poultry	Paterna	2016
Se_V_32	Pork	Valencia	2016
Se_V_33	Pork	Cheste	2016
Se_V_34	Pork	Valencia	2016
Se_V_35	Pork	Orihuela	2016
Se_V_36	Pork	Alicante	2016
Se_V_37	Pork	Valencia	2015
Se_V_38	Pork	Torrent	2015
Se_V_39	Pork	Valencia	2015
Se_V_40	Meat preparation	Torrent	2015
Se_V_41	Chicken-pork	Ibi	2016
Se_V_42	Pork	Vilajoyosa	2016
Se_V_43	Pork	Vilajoyosa	2016
Se_V_44	Pork	Pinoso	2016
Se_V_45	Pork	Valencia	2016
Se_V_46	Chicken	Elche	2016
Se_V_47	Pork	Torrent	2016

Se_V_48	Pork	Valencia	2016
Se_V_49	Chicken	Rafelbunyol	2016
Se_V_50	Chicken	Rafelbunyol	2016
Se_V_51	Poultry	Ibi	2017
Se_V_52	Poultry	Paterna	2017
Se_V_53	Chicken	Valencia	2017
Se_V_54	Chicken	Borriana	2017
Se_V_55	Chicken	Borriana	2017
Se_V_56	Chicken-pork	Paterna	2017
Se_V_57	Chicken-pork	Paterna	2017
Se_V_58	Poultry	Paterna	2017
Se_V_59	Mussel	Valencia	2015
Se_V_60	Tellina	Valencia	2015
Se_V_61	Egg	Enguera	2016
Se_V_62	Egg	Enguera	2016
Se_V_63	Poultry	Paterna	2017
Se_V_64	Humboldt squid	Ayora	2017
Se_V_65	Octopus	Torrent	2017
Se_V_66	Pork	Borriana	2017
Se_V_67	Pork	Valencia	2017
Se_V_68	Pork	Valencia	2017
Se_V_69	Pork	Castellón	2017
Se_V_70	Chicken	Alzira	2014
Se_V_71	Chicken	GaNAía	2015
Se_V_72	Chicken	Rafelbunyol	2016
Se_V_73	Mussel	Quart de Poblet	2016

Se_V_74	Mussel	Quart de Poblet.	2016
Se_V_75	Chicken	Valencia	2013
Se_V_76	Chicken	Alzira	2013
Se_V_77	Poultry	Ibi	2017
Se_V_78	Chicken	Algemesí	2017
Se_V_79	Feces	Artana	2017
Se_V_80	Burger meat	Denia	2013
Se_V_81	Chicken	Valencia	2014
Se_V_82	Pork	Denia	2015
Se_V_83	Ice cream	Castellón	2016
Se_V_84	Ice cream	Alcalà de Xivert	2016
Se_V_85	Salad	Valencia	2014
Se_V_86	Chicken	Valencia	2014
Se_V_87	Oysters	Valencia	2017
Se_V_88	Poultry	Elche	2017
Se_V_89	Pork	GaNAia	2017
Se_V_90	Russian salad	GaNAia	2017
Se_V_91	Chicken	Cox	2017
Se_V_92	Chicken	Cox	2017
Se_V_93	Mussel	Valencia	2015
Se_V_94	Turkey	Vall d´Uixo	2017
Se_V_95	Mussel	Utiel	2015
Se_V_96	Mussel	Torrent	2015
Se_V_97	Chicken	Almazora	2016
Se_V_98	Chicken	Sueca	2016
Se_V_99	Chicken	Valencia	2016

Se_V_100	Chicken	Sueca	2016
Se_V_101	Poultry	Benaguasil	2017
Se_V_102	Poultry	Ibi	2017
Se_V_103	Chicken	Sueca	2017
Se_V_104	Chicken	Sueca	2017
Se_V_105	Chicken	Sueca	2017
Se_V_106	Turtle aquarium	Castellón	2015
Se_V_107	Turkey	Torrent	2017
Se_V_108	Poultry	Alzira	2010
Se_V_109	Chicken	Torrent	2017
Se_V_110	Chicken	Torrent	2017
Se_V_111	Poultry	Algemesí	2017
Se_V_112	Burger meat	Alzira	2014
Se_V_113	Poultry	Ibi	2017
Se_V_114	Turkey	Valencia	2017
Se_V_115	Mussels	Puerto de Sagunto	2014
Se_V_116	Pork	Requena	2015
Se_V_117	Pork	Oliva	2016
Se_V_118	Pork	Valencia	2016
Se_V_119	Pork	Vila-real	2016
Se_V_120	Pork	Paterna	2017
Se_V_121	Pork	Betxí	2017
Se_V_122	Pork	Betxí	2015
Se_V_123	Pork	Oliva	2016
Se_V_124	Chicken	Almassora	2017
Se_V_125	Poultry	Paterna	2017

Se_V_126	Pork	Torrent	2017
Se_V_127	Black pepper	Novelda	2017
Se_V_128	Chicken	Valencia	2014
Se_V_129	Chicken	Ontinyent	2017
Se_V_130	Chicken	Almassora	2017
Se_V_131	Chicken	Alcalá de Xivert	2017
Se_V_133	Chicken	Almazora	2016
Se_V_134	Poultry	Almazora	2017
Se_V_135	Chicken	Castellón	2017
Se_V_137	Poultry	Almazora	2017
Se_V_138	Poultry	Almazora	2017
Se_V_139	Chicken	Valencia	2014
Se_V_140	Pork	Oliva	2016
Se_V_141	Russian salad	Elche	2017
Se_V_142	Chicken	Valencia	2017
Se_V_143	Poultry	Villajoyosa	2017

Table S2. Summary of sequence reads quality. Total reads, GC content, and average size obtained by FastQC and MultiQC from raw and cleaned reads.

Isolate	Raw reads			Cleaned reads		
	Total reads	%GC	Average reads length	Total reads	%GC	Average reads length
Se_V_1	1139667	51	138.6	987316	51	142.7
Se_V_2	1408524	52	132.4	1141553	52	141.4
Se_V_3	1003432	51	140.1	879671	51	144.7
Se_V_4	1251382	51	138.6	1087265	51	142.9
Se_V_5	1046550	51	140.3	921255	51	143.6
Se_V_6	964128	51	140.2	845341	51	143.4
Se_V_7	1301591	51	140.6	1148838	51	145
Se_V_8	1107342	51	140.3	972551	51	143.4
Se_V_9	1052415	51	139.2	919091	51	144.2
Se_V_10	821588	51	141	724793	51	145.2
Se_V_11	936900	52	140.2	821726	52	143.5
Se_V_12	1190982	51	140.4	1048466	51	143.5
Se_V_13	1083850	51	140.4	953266	51	143.5
Se_V_14	704363	51	140.8	620981	51	143.5
Se_V_15	521349	51	141.1	459680	51	143.5
Se_V_16	680095	51	140.9	600798	51	143.6
Se_V_17	621269	51	141.1	550733	51	145.2
Se_V_18	790312	51	140.1	692343	51	143.4
Se_V_19	845666	51	140.3	744709	51	143.5
Se_V_20	559338	50	141.1	493990	50	145.2
Se_V_21	255617	51	140	221230	51	142.8
Se_V_22	339834	51	140.2	295608	51	143
Se_V_23	466442	51	141.1	410232	51	145.2
Se_V_24	835379	51	140.7	737709	51	143.6
Se_V_25	1229079	51	140.1	1064186	51	145

Se_V_26	839511	51	141.2	737611	51	143.4
Se_V_27	817609	51	139.7	713375	51	142.9
Se_V_28	667046	51	139.9	586019	50	143.3
Se_V_29	810866	51	140.4	714596	51	144.8
Se_V_30	842257	51	140.1	736721	51	143.2
Se_V_31	652853	51	140.6	574687	51	143.4
Se_V_32	557980	51	140.5	489026	51	143.2
Se_V_33	660750	51	140.7	579385	51	145
Se_V_34	979419	51	140.8	855865	51	145.2
Se_V_35	662939	51	140.7	583128	51	145
Se_V_36	443679	51	141.2	389564	51	143.4
Se_V_37	530421	51	140.9	465795	51	145.1
Se_V_38	542729	50	141.4	476986	51	143.4
Se_V_39	435085	51	140.7	379715	51	143.2
Se_V_40	519044	51	140.9	456814	51	145.1
Se_V_41	579587	51	141	511113	51	145.2
Se_V_42	416745	51	140.5	363241	51	143.2
Se_V_43	623817	51	140.5	547978	51	143.4
Se_V_44	707599	50	140.4	619864	50	143.1
Se_V_45	509773	51	140.1	442295	51	143
Se_V_46	750250	51	141.1	658471	51	145.3
Se_V_47	648853	51	141	570698	51	145.2
Se_V_48	896645	51	140.5	788137	51	144.9
Se_V_49	680849	51	140.7	596172	51	145.1
Se_V_50	587468	51	140.9	512612	51	145.3
Se_V_51	546426	51	141	477219	51	143.1
Se_V_52	122931	51	136.7	98729	51	139.3
Se_V_53	598487	51	141.1	527598	51	145.2
Se_V_54	714899	51	139.9	624302	51	143.1
Se_V_55	714485	51	140.4	628023	51	144.8
Se_V_56	652154	51	141.3	573281	51	143.4
Se_V_57	470682	51	140.4	408591	51	142.9
Se_V_58	766558	51	140.6	673289	51	143.3

Se_V_59	968497	51	139.5	844305	51	144.4
Se_V_60	775645	51	141	681402	51	145.2
Se_V_61	285532	51	137.5	240877	51	144
Se_V_62	648792	51	140.8	573124	51	145
Se_V_63	565476	51	140.2	495881	51	143.4
Se_V_64	622697	51	140.1	547338	51	144.7
Se_V_65	732076	51	140.1	643824	51	143.5
Se_V_66	493959	50	140	432314	50	143.4
Se_V_67	606542	51	140.4	535938	51	143.5
Se_V_68	553615	51	139.7	485332	51	143.2
Se_V_69	655706	50	140.1	576904	50	143.4
Se_V_70	687746	51	140.9	607268	51	143.6
Se_V_71	682219	51	140.6	602767	51	143.6
Se_V_72	562931	51	140.1	492373	51	143.5
Se_V_73	993753	50	140.2	867904	50	144.8
Se_V_74	765436	50	140.5	669707	50	145
Se_V_75	672470	51	140.8	592399	51	143.4
Se_V_76	866922	51	141.1	765432	51	145.1
Se_V_77	695033	51	139.8	607761	51	144.6
Se_V_78	688998	51	139.9	601107	51	144.7
Se_V_79	697385	49	140.4	615555	49	143.3
Se_V_80	597210	51	140.4	523903	51	144.8
Se_V_81	655654	51	140.1	573769	51	143.3
Se_V_82	755425	51	140	658714	51	143.3
Se_V_83	667674	50	140.2	586462	50	143.3
Se_V_84	405494	51	140.5	353075	51	145.1
Se_V_85	1031298	51	137.8	885946	51	143.4
Se_V_86	876157	51	139.1	760869	51	144.1
Se_V_87	692093	51	137	590131	51	143.1
Se_V_88	912150	51	138	786241	51	143.5
Se_V_89	1078883	51	135.6	907734	51	141.4
Se_V_90	873094	50	137.3	746590	50	143.2
Se_V_91	924317	51	140.3	809075	51	143.3

Se_V_92	810655	51	139.5	706307	51	142.8
Se_V_93	894289	50	138.7	775062	50	143.9
Se_V_94	1045271	51	136.5	878619	51	141
Se_V_95	733977	50	139.4	638273	50	144.4
Se_V_96	749790	50	140.5	657060	50	144.9
Se_V_97	965628	51	137.6	831025	51	142.3
Se_V_98	719212	51	139.4	628596	51	143.1
Se_V_99	704334	51	140.2	619223	51	144.7
Se_V_100	780697	51	137.9	673717	51	142.4
Se_V_101	798672	51	138.9	696018	51	144
Se_V_102	688129	51	139.3	599974	51	142.9
Se_V_103	718971	51	139.7	629171	51	143.1
Se_V_104	752761	51	139.3	657781	51	143
Se_V_105	695050	51	139.1	606599	51	142.9
Se_V_106	765819	51	138.4	663701	51	143.8
Se_V_107	882002	51	136.8	751697	51	141.9
Se_V_108	775744	51	139.7	680928	51	144.4
Se_V_109	1085497	51	139.6	952022	51	143.2
Se_V_110	1108411	51	139.3	968967	51	144.3
Se_V_111	744028	51	140.6	657219	51	144.9
Se_V_112	528772	51	139.5	463957	51	143.2
Se_V_113	121061	47	133.7	99119	47	142.1
Se_V_114	724506	51	139.2	631996	51	142.9
Se_V_115	723497	51	139.7	634078	51	144.4
Se_V_116	1312337	51	125.6	955825	51	139.6
Se_V_117	764275	51	139.6	670596	51	144.4
Se_V_118	645558	51	140.4	569025	51	144.8
Se_V_119	831233	51	138.9	724098	51	143.9
Se_V_120	711062	51	138.8	620366	51	144
Se_V_121	747966	51	139.9	655523	51	144.6
Se_V_122	685841	51	138.7	593208	51	144.2
Se_V_123	501970	51	128.9	381161	51	141.5
Se_V_124	251273	51	135.6	205033	51	143.6

Se_V_125	333563	50	127.4	248579	51	140.9
Se_V_126	639141	51	128.5	482783	51	140.1
Se_V_127	638094	51	128.8	485005	51	140.2
Se_V_128	840777	51	137	714893	51	143.4
Se_V_129	156710	50	129.2	119797	50	141.6
Se_V_130	178516	51	133.7	141559	51	143
Se_V_131	704258	51	139.6	613979	51	144.4
Se_V_132	5525353	51	140.2	4848056	51	143.3
Se_V_133	1008020	51	138.8	874152	51	143
Se_V_134	516870	51	138.6	448135	51	142.7
Se_V_135	664461	51	140.5	584541	51	143.4
Se_V_136	250151	51	136.4	212581	51	141.7
Se_V_137	1270646	51	138.4	1101105	51	143.8
Se_V_138	876103	51	139.6	766035	51	144.4
Se_V_139	1067589	51	138.1	920941	51	142.4
Se_V_140	1170019	51	139.3	1021575	51	144.3
Se_V_141	883741	51	139.3	771011	51	143
Se_V_142	1978134	51	137.3	1693586	51	143.3
Se_V_143	735033	51	139.6	636490	51	142.6
Average	777649.20	50.88	139.14	675013.98	50.888	143.58

Table S3. Summary of assembly results. Number of contigs, length of the longest contig, total length aNA N50 value obtained by QUAST.

Isolate	Contigs	Longest contig	Total length	N50
Se_V_1	60	419299	4778074	207005
Se_V_2	29	1583715	4748065	536785
Se_V_3	43	448471	4643747	218737
Se_V_4	34	709476	4677025	485480
Se_V_5	36	551910	4677150	393692
Se_V_6	41	459444	4679304	244336
Se_V_7	46	595042	4790469	226874
Se_V_8	47	549298	4789798	226976
Se_V_9	42	580763	4828211	251737
Se_V_10	36	809006	4828819	319611
Se_V_11	49	809420	4915671	292067
Se_V_12	40	809055	4830964	327334
Se_V_13	80	608352	4931017	223101
Se_V_14	80	474809	4902966	199854
Se_V_15	117	350759	5005313	184842
Se_V_16	76	580564	4903295	223164
Se_V_17	123	442843	4949491	114881
Se_V_18	87	442859	4897190	224944
Se_V_19	126	516285	4971738	172402
Se_V_20	90	547484	4932352	246881
Se_V_21	117	435617	4985937	109509
Se_V_22	70	362507	4881704	217595
Se_V_23	56	663447	4880756	274418
Se_V_24	51	851312	4817831	269940
Se_V_25	119	443139	5169747	165836
Se_V_26	113	547667	4933955	162257
Se_V_27	111	547543	4932033	201130
Se_V_28	88	376901	4909679	267192
Se_V_29	88	435934	4911057	254459

Se_V_30	118	442901	4933701	162256
Se_V_31	85	435843	4906109	247080
Se_V_32	63	419297	4777483	206912
Se_V_33	136	464312	4942020	165696
Se_V_34	91	504781	4904465	199854
Se_V_35	83	443039	4874509	223701
Se_V_36	84	548478	4967285	247081
Se_V_37	101	474938	4970561	255535
Se_V_38	89	435655	5018376	222750
Se_V_39	92	442856	5015610	166917
Se_V_40	102	548327	5077105	262315
Se_V_41	99	474886	5077702	278554
Se_V_42	92	376701	4766910	173871
Se_V_43	94	276732	4768899	129624
Se_V_44	109	474906	5135382	214967
Se_V_45	83	442901	4968250	256254
Se_V_46	47	809001	4915836	302524
Se_V_47	85	307510	4953467	199726
Se_V_48	83	495479	4913649	223101
Se_V_49	68	590265	4806169	183687
Se_V_50	70	443296	4805015	149697
Se_V_51	67	438969	4914310	212653
Se_V_52	1475	21717	4752118	4678
Se_V_53	69	438876	4806720	196848
Se_V_54	69	438968	4903188	183946
Se_V_55	64	476580	4903561	181036
Se_V_56	72	438968	4915751	203642
Se_V_57	78	438782	4913830	183687
Se_V_58	73	438782	4916190	180989
Se_V_59	119	547212	4863951	223101
Se_V_60	121	442903	4864180	154741
Se_V_61	94	486454	4871035	130142
Se_V_62	71	448901	4872365	202147

Se_V_63	94	545931	4982610	175429
Se_V_64	76	486880	4873284	202147
Se_V_65	75	486633	4872597	208887
Se_V_66	99	393816	4941112	213860
Se_V_67	89	576972	4954791	224779
Se_V_68	86	472938	4937568	223580
Se_V_69	94	443099	4940690	246881
Se_V_70	38	812655	4696091	307641
Se_V_71	64	438876	4805865	203915
Se_V_72	71	438969	4805860	152221
Se_V_73	99	225498	4903475	105759
Se_V_74	102	285506	4909793	127449
Se_V_75	45	830960	4684645	245380
Se_V_76	38	610159	4686310	400657
Se_V_77	59	554208	5010270	285317
Se_V_78	49	544596	4687157	245382
Se_V_79	197	210772	5056615	68068
Se_V_80	67	377417	4938982	152724
Se_V_81	80	377215	4834885	170311
Se_V_82	81	349439	4927717	192780
Se_V_83	94	601794	4940867	223164
Se_V_84	74	464304	4871042	178209
Se_V_85	41	973978	4698098	229237
Se_V_86	41	973630	4697607	229237
Se_V_87	44	650786	4689048	229199
Se_V_88	44	650923	4697729	229237
Se_V_89	43	933336	4689529	227918
Se_V_90	249	392253	9905380	98311
Se_V_91	39	650871	4695487	249841
Se_V_92	43	650773	4695785	249846
Se_V_93	96	373219	5095404	148920
Se_V_94	64	635792	4749015	188352
Se_V_95	99	285318	4908446	127449

Se_V_96	98	285505	4903627	115293
Se_V_97	43	809370	4787985	327334
Se_V_98	43	580761	4819169	256432
Se_V_99	46	580763	4802268	292067
Se_V_100	44	580706	4801249	304280
Se_V_101	41	809029	4819991	292067
Se_V_102	42	580332	4820027	302524
Se_V_103	44	580761	4818681	251738
Se_V_104	46	580761	4813026	256425
Se_V_105	39	580763	4813890	304547
Se_V_106	52	418337	4728423	200018
Se_V_107	34	1475733	4604584	289228
Se_V_108	39	770066	4674245	264656
Se_V_109	46	382017	4630839	226523
Se_V_110	42	402876	4629661	226523
Se_V_111	42	488909	4724421	226009
Se_V_112	59	342100	4575932	165475
Se_V_113	3133	8706	3226157	1103
Se_V_114	50	402876	4733990	217735
Se_V_115	54	560409	4897638	190285
Se_V_116	48	560462	4863491	293243
Se_V_117	59	495918	4985684	190285
Se_V_118	59	429423	4863821	188685
Se_V_119	79	560320	4931450	190285
Se_V_120	59	451841	4860302	188848
Se_V_121	55	495765	4935629	239685
Se_V_122	124	442893	5025529	135128
Se_V_123	66	644840	4708840	276576
Se_V_124	73	467539	4700018	152356
Se_V_125	117	194107	4847427	86835
Se_V_126	52	488705	4886522	188693
Se_V_127	44	391883	4555036	234881
Se_V_128	54	551502	4622361	225887

Se_V_129	1379	28381	4627796	5041
Se_V_130	684	59526	5182016	14108
Se_V_131	57	567931	5250158	233305
Se_V_133	33	546545	4631386	263630
Se_V_134	40	860033	4629660	232301
Se_V_135	34	481488	4629387	308383
Se_V_137	36	522729	4630020	263630
Se_V_138	37	481838	4629741	232301
Se_V_139	50	564546	4716606	231378
Se_V_140	67	442032	4802909	172766
Se_V_141	59	539393	4824496	243067
Se_V_142	48	564668	4723807	235055
Se_V_143	73	438969	4915686	196848
Average	116.58	518125.60	4868712.02	216087.23

Table S4. Serotypes obtained by serology, SeqSero2 and SISTR and ST obtained by Sanger sequencing and WGS. PMV: Potential monophasic variant of Typhimurium.

Isolate	Serology	SeqSero2	SISTR	ST Sanger	ST WGS
Se_V_1	Derby	Derby	Derby	40	40
Se_V_2	Anatum	Anatum	Anatum	64	64
Se_V_3	Schleissheim	Paratyphi B	Paratyphi B	127	127
Se_V_4	Bovismorbificans	Bovismorbificans	Bovismorbificans	142	142
Se_V_5	Bovismorbificans	Bovismorbificans	Bovismorbificans	142	142
Se_V_6	LoNAon	LoNAon	LoNAon	155	155
Se_V_7	Newport	Newport	Newport	166	166
Se_V_8	Newport	Newport	Newport	166	166
Se_V_9	Kentucky	Kentucky	Kentucky	198	198
Se_V_10	Kentucky	Kentucky	Kentucky	198	198
Se_V_11	Kentucky	Kentucky	Kentucky	198	198
Se_V_12	Kentucky	Kentucky	Kentucky	198	198
Se_V_13	Typhimurium	Typhimurium (PMV)	Typhimurium (PMV)	34	34
Se_V_14	Typhimurium	Typhimurium	Typhimurium	34	34
Se_V_15	Typhimurium	Typhimurium (PMV)	Typhimurium (PMV)	34	34
Se_V_16	Typhimurium	Typhimurium	Typhimurium	34	34
Se_V_17	Typhimurium	Typhimurium (PMV)	Typhimurium (PMV)	34	34
Se_V_18	Typhimurium	Typhimurium	Typhimurium	34	34
Se_V_19	Typhimurium	Typhimurium (PMV)	Typhimurium (PMV)	34	34
Se_V_20	Typhimurium	Typhimurium (PMV)	Typhimurium (PMV)	34	34
Se_V_21	Typhimurium	Typhimurium	Typhimurium	34	19
Se_V_22	Derby	Derby	Derby	40	40
Se_V_23	Derby	Derby	Derby	40	40
Se_V_24	Derby	Derby	Derby	40	40

Se_V_25	Typhimurium	Typhimurium (PMV)	Typhimurium (PMV)	34	34
Se_V_26	Typhimurium	Typhimurium (PMV)	Typhimurium (PMV)	34	34
Se_V_27	Typhimurium	Typhimurium (PMV)	Typhimurium (PMV)	34	34
Se_V_28	Typhimurium	Typhimurium (PMV)	Typhimurium (PMV)	34	34
Se_V_29	Typhimurium	Typhimurium (PMV)	Typhimurium (PMV)	34	34
Se_V_30	Typhimurium	Typhimurium (PMV)	Typhimurium (PMV)	34	34
Se_V_31	Typhimurium	Typhimurium (PMV)	Typhimurium (PMV)	34	34
Se_V_32	Derby	Derby	Derby	40	40
Se_V_33	Typhimurium	Typhimurium (PMV)	Typhimurium (PMV)	34	34
Se_V_34	Typhimurium	Typhimurium (PMV)	Typhimurium (PMV)	34	34
Se_V_35	Typhimurium	Typhimurium (PMV)	Typhimurium (PMV)	34	34
Se_V_36	Typhimurium	Typhimurium (PMV)	Typhimurium (PMV)	34	34
Se_V_37	Typhimurium	Typhimurium (PMV)	Typhimurium (PMV)	34	34
Se_V_38	Typhimurium	Typhimurium (PMV)	Typhimurium (PMV)	34	34
Se_V_39	Typhimurium	Typhimurium (PMV)	Typhimurium (PMV)	34	34
Se_V_40	Typhimurium	Typhimurium (PMV)	Typhimurium (PMV)	34	34
Se_V_41	Typhimurium	Typhimurium (PMV)	Typhimurium (PMV)	34	34
Se_V_42	Typhimurium	Typhimurium	Typhimurium	34	34
Se_V_43	Typhimurium	Typhimurium	Typhimurium	34	34
Se_V_44	Typhimurium	Typhimurium (PMV)	Typhimurium (PMV)	34	34
Se_V_45	Typhimurium	Typhimurium (PMV)	Typhimurium (PMV)	34	34
Se_V_46	Kentucky	Kentucky	Kentucky	198	198
Se_V_47	Typhimurium	Typhimurium (PMV)	Typhimurium (PMV)	34	34
Se_V_48	Typhimurium	Typhimurium (PMV)	Typhimurium (PMV)	34	34
Se_V_49	Infantis	Infantis	Infantis	32	32
Se_V_50	Infantis	Infantis	Infantis	32	32
Se_V_51	Infantis	Infantis	Infantis	32	32

Se_V_53	Infantis	Infantis	Infantis	32	32
Se_V_54	Infantis	Infantis	Infantis	32	32
Se_V_55	Infantis	Infantis	Infantis	32	32
Se_V_56	Infantis	Infantis	Infantis	32	32
Se_V_57	Infantis	Infantis	Infantis	32	32
Se_V_58	Infantis	Infantis	Infantis	32	32
Se_V_59	Typhimurium	Typhimurium (PMV)	Typhimurium (PMV)	34	34
Se_V_60	Typhimurium	Typhimurium (PMV)	Typhimurium (PMV)	34	34
Se_V_61	Typhimurium	Typhimurium	Typhimurium	19	19
Se_V_62	Typhimurium	Typhimurium	Typhimurium	19	19
Se_V_63	Typhimurium	Typhimurium	Typhimurium	19	19
Se_V_64	Typhimurium	Typhimurium	Typhimurium	19	19
Se_V_65	Typhimurium	Typhimurium	Typhimurium	19	19
Se_V_66	Typhimurium	Typhimurium (PMV)	Typhimurium (PMV)	19	34
Se_V_67	Typhimurium	Typhimurium (PMV)	Typhimurium (PMV)	19	34
Se_V_68	Typhimurium	Typhimurium (PMV)	Typhimurium (PMV)	19	34
Se_V_69	Typhimurium	Typhimurium (PMV)	Typhimurium (PMV)	19	34
Se_V_70	Thompson	Thompson	Thompson	26	26
Se_V_71	Infantis	Infantis	Infantis	32	32
Se_V_72	Infantis	Infantis	Infantis	32	32
Se_V_73	Senftenberg	Senftenberg	Senftenberg	14	14
Se_V_74	Senftenberg	Senftenberg	Senftenberg	14	14
Se_V_75	Virchow	Virchow	Virchow	16	16
Se_V_76	Virchow	Virchow	Virchow	16	16
Se_V_77	Virchow	Virchow	Virchow	16	16
Se_V_78	Virchow	Virchow	Virchow	16	16
Se_V_80	Typhimurium	Typhimurium	Typhimurium	19	19

Se_V_81	Typhimurium	Typhimurium	Typhimurium	19	19
Se_V_82	Typhimurium	Typhimurium	Typhimurium	19	19
Se_V_83	Typhimurium	Typhimurium (PMV)	Typhimurium (PMV)	19	34
Se_V_84	Typhimurium	Typhimurium	Typhimurium	19	19
Se_V_85	Enteritidis	Enteritidis	Enteritidis	11	11
Se_V_86	Enteritidis	Enteritidis	Enteritidis	11	11
Se_V_87	Enteritidis	Enteritidis	Enteritidis	11	11
Se_V_88	Enteritidis	Enteritidis	Enteritidis	11	11
Se_V_89	Enteritidis	Enteritidis	Enteritidis	11	11
Se_V_91	Enteritidis	Enteritidis	Enteritidis	11	11
Se_V_92	Enteritidis	Enteritidis	Enteritidis	11	11
Se_V_93	Agona	Agona	Agona	13	13
Se_V_94	Agona	Agona	Agona	13	13
Se_V_95	Senftenberg	Senftenberg	Senftenberg	14	14
Se_V_96	Senftenberg	Senftenberg	Senftenberg	14	14
Se_V_97	Kentucky	Kentucky	Kentucky	198	198
Se_V_98	Kentucky	Kentucky	Kentucky	198	198
Se_V_99	Kentucky	Kentucky	Kentucky	198	198
Se_V_100	Kentucky	Kentucky	Kentucky	198	198
Se_V_101	Kentucky	Kentucky	Kentucky	198	198
Se_V_102	Kentucky	Kentucky	Kentucky	198	198
Se_V_103	Kentucky	Kentucky	Kentucky	198	198
Se_V_104	Kentucky	Kentucky	Kentucky	198	198
Se_V_105	Kentucky	Kentucky	Kentucky	198	198
Se_V_106	Litchfield	Litchfield	Litchfield	214	214
Se_V_107	Bredeney	Bredeney	Bredeney	241	241
Se_V_108	Bredeney	Bredeney	Bredeney	306	306

Se_V_109	Bredeney	Bredeney	Bredeney	306	306
Se_V_110	Bredeney	Bredeney	Bredeney	306	306
Se_V_111	Montevideo	Montevideo	Montevideo	316	316
Se_V_112	Brandenburg	Brandenburg	Brandenburg	334	334
Se_V_114	Bredeney	Bredeney	Bredeney	306	306
Se_V_115	Rissen	Rissen	Rissen	469	469
Se_V_116	Rissen	Rissen	Rissen	469	469
Se_V_117	Rissen	Rissen	Rissen	469	469
Se_V_118	Rissen	Rissen	Rissen	469	469
Se_V_119	Rissen	Rissen	Rissen	469	469
Se_V_120	Rissen	Rissen	Rissen	469	469
Se_V_121	Rissen	Rissen	Rissen	469	469
Se_V_122	Typhimurium	Typhimurium (PMV)	Typhimurium (PMV)	34	34
Se_V_123	Derby	Derby	Derby	682	682
Se_V_124	Cerro	Cerro	Cerro	1291	1291
Se_V_125	Rissen	Rissen	Rissen	469	469
Se_V_126	Rissen	Rissen	Rissen	469	469
Se_V_127	Rubislaw	Rubislaw	Rubislaw	1575	1575
Se_V_128	Cerro	Cerro	Cerro	1593	1593
Se_V_131	Mikawasima	Mikawasima	Mikawasima	1815	1815
Se_V_133	Chester	Chester	Chester	1954	1954
Se_V_134	Chester	Chester	Chester	1954	1954
Se_V_135	Chester	Chester	Chester	1954	1954
Se_V_137	Chester	Chester	Chester	1954	1954
Se_V_138	Chester	Chester	Chester	1954	1954
Se_V_139	Senftenberg	Llandoff	Jedburgh|Llandoff	New	5689
Se_V_140	NA	Typhimurium	Typhimurium	New	5237

Se_V_141	NA	Mendoza	Kapemba|Miyazaki	New	5104
Se_V_142	NA	Llandoff	Jedburgh|Llandoff	New	5689
Se_V_143	NA	Infantis	Infantis	New	32

Table S5. High similarity clusters (HSC) isolates according to the threshold levels selected: 10, 5 and 3 SNPs. The product, place and year of sampling for each isolate are shown. The table also shows the resistance genes identified, the inferred antimicrobial resistance phenotype from these and from the estimated MIC values, and the detected plasmid replicon types.

Isolate	Product	Place	Year	Serology	SeqSero2	SISTR	ST_ WGS	Cluster (10 SNPs)	Cluster (5 SNPs)	Cluster (3 SNPs)	Genes	Predicted Resistant Phenotype (genes)	AMR predicted from inferred MICs	Plasmid replicon types
Se_V_101	Poultry	Benaguasil	2017	Kentucky	Kentucky	Kentucky	198	1	27	65	gyrA (D87N), gyrA (S83F)	ciprofloxacin I/R, nalidixic acid	streptomycin	None
Se_V_102	Poultry	Ibi	2017	Kentucky	Kentucky	Kentucky	198	1	27	65	gyrA (D87N), gyrA (S83F)	ciprofloxacin I/R, nalidixic acid	streptomycin	None
Se_V_98	Chicken	Sueca	2016	Kentucky	Kentucky	Kentucky	198	1	27	65	gyrA (D87N), gyrA (S83F)	ciprofloxacin I/R, nalidixic acid	streptomycin	None
Se_V_10	Chicken	Denia	2013	Kentucky	Kentucky	Kentucky	198	1	29	67	gyrA (D87N), gyrA (S83F)	ciprofloxacin I/R, nalidixic acid	streptomycin	None
Se_V_9	Chicken	Denia	2013	Kentucky	Kentucky	Kentucky	198	1	29	67	gyrA (D87N), gyrA (S83F)	ciprofloxacin I/R, nalidixic acid	streptomycin	None
Se_V_104	Chicken	Sueca	2017	Kentucky	Kentucky	Kentucky	198	1	40	78	gyrA (D87N), gyrA (S83F)	ciprofloxacin I/R, nalidixic acid	streptomycin	None
Se_V_105	Chicken	Sueca	2017	Kentucky	Kentucky	Kentucky	198	1	40	78	gyrA (D87N), gyrA (S83F)	ciprofloxacin I/R, nalidixic acid	streptomycin	None
Se_V_103	Chicken	Sueca	2017	Kentucky	Kentucky	Kentucky	198	1	40	96	gyrA (D87N), gyrA (S83F)	ciprofloxacin I/R, nalidixic acid	streptomycin	None
Se_V_73	Mussel	Quart	2016	Senftenberg	Senftenberg	Senftenberg	14	2	28	66	None	Sensitive	streptomycin	IncR
Se_V_74	Mussel	Quart	2016	Senftenberg	Senftenberg	Senftenberg	14	2	28	66	None	Sensitive	streptomycin	IncR
Se_V_95	Mussel	Utiel	2015	Senftenberg	Senftenberg	Senftenberg	14	2	28	66	None	Sensitive	streptomycin	IncR
Se_V_96	Mussel	Torrent	2015	Senftenberg	Senftenberg	Senftenberg	14	2	28	66	None	Sensitive	streptomycin	IncR
Se_V_66	Pork	Borriana	2017	Typhimurium	Typhimurium (PMV)	Typhimurium (PMV)	34	3	30	68	aph(3")-Ib, aph(6)-Id, blaTEM-1B, sul2, tet(B)	streptomycin, kanamycin, ampicillin, sulfisoxazole, tetracycline	ampicillin sulfisoxazole streptomycin, tetracycline,	IncQ1
Se_V_69	Pork	Castellón	2017	Typhimurium	Typhimurium (PMV)	Typhimurium (PMV)	34	3	30	68	aph(3")-Ib, aph(6)-Id, blaTEM-1B, sul2, tet(B)	streptomycin, kanamycin, ampicillin, sulfisoxazole, tetracycline	streptomycin, tetracycline,	IncQ1
Se_V_83	Ice cream	Castellón	2016	Typhimurium	Typhimurium (PMV)	Typhimurium (PMV)	34	3	30	68	aph(3")-Ib, aph(6)-Id, blaTEM-1B, sul2, tet(B)	streptomycin, kanamycin, ampicillin, sulfisoxazole, tetracycline	ampicillin sulfisoxazole streptomycin, tetracycline,	IncQ1
Se_V_20	Chicken	Torrent	2017	Typhimurium	Typhimurium (PMV)	Typhimurium (PMV)	34	3	49	88	aph(3")-Ib, aph(6)-Id, blaTEM-1B, sul2, tet(B)	streptomycin, kanamycin, ampicillin, sulfisoxazole, tetracycline	ampicillin sulfisoxazole streptomycin, tetracycline,	IncQ1
Se_V_38	Pork	Torrent	2015	Typhimurium	Typhimurium (PMV)	Typhimurium (PMV)	34	3	49	88	aph(3")-Ib, aph(6)-Id, blaTEM-1B, sul2, tet(B)	streptomycin, kanamycin, ampicillin, sulfisoxazole, tetracycline	ampicillin streptomycin, tetracycline,	IncQ1
Se_V_7	Poultry	Font Carrós	2016	Newport	Newport	Newport	166	4	31	69	None	Sensitive		None
Se_V_8	Poultry	Font Carrós	2016	Newport	Newport	Newport	166	4	31	69	None	Sensitive		None
Se_V_49	Chicken	Rafelbunyol	2016	Infantis	Infantis	Infantis	32	5	32	70	gyrA (S83Y)	ciprofloxacin I/R, nalidixic acid		None
Se_V_53	Chicken	Valencia	2017	Infantis	Infantis	Infantis	32	5	32	70	gyrA (S83Y)	ciprofloxacin I/R, nalidixic acid		None
Se_V_71	Chicken	Gandía	2015	Infantis	Infantis	Infantis	32	5	32	70	gyrA (S83Y)	ciprofloxacin I/R, nalidixic acid		None
Se_V_72	Chicken	Rafelbunyol	2016	Infantis	Infantis	Infantis	32	5	32	70	gyrA (S83Y)	ciprofloxacin I/R, nalidixic acid		None
Se_V_50	Chicken	Rafelbunyol	2016	Infantis	Infantis	Infantis	32	5	55	99	gyrA (S83Y)	ciprofloxacin I/R, nalidixic acid		None
Se_V_62	Egg	Enguera	2016	Typhimurium	Typhimurium	Typhimurium	19	6	33	71	None	Sensitive	streptomycin	IncFIB(S), IncFII(S)
Se_V_64	Squid	Ayora	2017	Typhimurium	Typhimurium	Typhimurium	19	6	33	71	None	Sensitive	streptomycin	IncFIB(S), IncFII(S)
Se_V_65	Octopus	Torrent	2017	Typhimurium	Typhimurium	Typhimurium	19	6	33	71	None	Sensitive	streptomycin	IncFIB(S), IncFII(S)
Se_V_84	Ice cream	Alcalà Xivert	2016	Typhimurium	Typhimurium	Typhimurium	19	6	33	71	None	Sensitive	streptomycin	IncFIB(S), IncFII(S)
Se_V_35	Pork	Orihuela	2016	Typhimurium	Typhimurium (PMV)	Typhimurium (PMV)	34	7	34	72	aph(3")-Ib, aph(6)-Id, blaTEM-1B, sul2, tet(B)	streptomycin, kanamycin, ampicillin, sulfisoxazole, tetracycline	streptomycin, tetracycline,	IncQ1
Se_V_59	Mussel	Valencia	2015	Typhimurium	Typhimurium (PMV)	Typhimurium (PMV)	34	7	34	72	aph(3")-Ib, aph(6)-Id, blaTEM-1B, sul2, tet(B)	streptomycin, kanamycin, ampicillin, sulfisoxazole, tetracycline	streptomycin, tetracycline,	IncQ1
Se_V_60	Clam	Valencia	2015	Typhimurium	Typhimurium (PMV)	Typhimurium (PMV)	34	7	34	72	aph(3")-Ib, aph(6)-Id, blaTEM-1B, sul2, tet(B)	streptomycin, kanamycin, ampicillin, sulfisoxazole, tetracycline	streptomycin, tetracycline,	IncQ1
Se_V_26	Pork	Sueca	2016	Typhimurium	Typhimurium (PMV)	Typhimurium (PMV)	34	7	34	86	aph(3")-Ib, aph(6)-Id, blaTEM-1B, sul2, tet(B)	streptomycin, kanamycin, ampicillin, sulfisoxazole, tetracycline	streptomycin, tetracycline,	IncQ1

Se_V_27	Pork	Sueca	2016	Typhimurium	Typhimurium (PMV)	Typhimurium (PMV)	34	7	34	86	aph(3'')-Ib, aph(6)-Id, blaTEM-1B, sul2, tet(B)	streptomycin, kanamycin, ampicillin, sulfisoxazole, tetracycline	streptomycin, tetracycline,	IncQ1
Se_V_30	Pork	Valencia	2016	Typhimurium	Typhimurium (PMV)	Typhimurium (PMV)	34	7	34	95	aph(3'')-Ib, aph(6)-Id, blaTEM-1B, sul2, tet(B)	streptomycin, kanamycin, ampicillin, sulfisoxazole, tetracycline	streptomycin, tetracycline,	IncQ1
Se_V_40	Meat preparation	Torrent	2015	Typhimurium	Typhimurium (PMV)	Typhimurium (PMV)	34	7	57	101	aadA1, aadA2, aph(3'')-Ib, aph(6)-Id, blaTEM-1B, cmlA1, dfrA12, floR, qnrB19, sul2, tet(B)	streptomycin, kanamycin, ampicillin, chloramphenicol, trimethoprim, ciprofloxacin I/R, sulfisoxazole, tetracycline	ampicillin sulfisoxazole streptomycin, tetracycline,	IncQ1, p0111
Se_V_15	Pork	Betxí	2017	Typhimurium	Typhimurium (PMV)	Typhimurium (PMV)	34	7	58	102	aph(3'')-Ib, aph(6)-Id, blaTEM-1B, sul2	streptomycin, kanamycin, ampicillin, sulfisoxazole	streptomycin	IncQ1, p0111
Se_V_19	Pasta salad	Benidorm	2017	Typhimurium	Typhimurium (PMV)	Typhimurium (PMV)	34	7	61	105	aph(3'')-Ib, aph(6)-Id, blaTEM-1B, sul2, tet(B)	streptomycin, kanamycin, ampicillin, sulfisoxazole, tetracycline	streptomycin, tetracycline,	IncQ1
Se_V_143	Poultry	Villajoyosa	2017	NA	Infantis	Infantis	32	8	35	73	ant(3'')-Ia, dfrA14, gyrA (S83Y), sul1, tet(A)	streptomycin, trimethoprim, ciprofloxacin I/R, nalidixic acid, sulfisoxazole, tetracycline	streptomycin, tetracycline,	None
Se_V_51	Poultry	Ibi	2017	Infantis	Infantis	Infantis	32	8	35	73	ant(3'')-Ia, dfrA14, gyrA (S83Y), sul1, tet(A)	streptomycin, trimethoprim, ciprofloxacin I/R, nalidixic acid, sulfisoxazole, tetracycline	streptomycin, tetracycline,	None
Se_V_56	Chicken-pork	Paterna	2017	Infantis	Infantis	Infantis	32	8	35	73	ant(3'')-Ia, dfrA14, gyrA (S83Y), sul1, tet(A)	streptomycin, trimethoprim, ciprofloxacin I/R, nalidixic acid, sulfisoxazole, tetracycline	streptomycin, tetracycline,	None
Se_V_57	Chicken-pork	Paterna	2017	Infantis	Infantis	Infantis	32	8	35	73	ant(3'')-Ia, dfrA14, gyrA (S83Y), sul1, tet(A)	streptomycin, trimethoprim, ciprofloxacin I/R, nalidixic acid, sulfisoxazole, tetracycline	streptomycin, tetracycline,	None
Se_V_58	Poultry	Paterna	2017	Infantis	Infantis	Infantis	32	8	35	73	ant(3'')-Ia, dfrA14, gyrA (S83Y), sul1, tet(A)	streptomycin, trimethoprim, ciprofloxacin I/R, nalidixic acid, sulfisoxazole, tetracycline	streptomycin, tetracycline,	None
Se_V_11	Chicken	Elche	2016	Kentucky	Kentucky	Kentucky	198	9	36	74	aac(3)-Id, aadA7, gyrA (D87N), gyrA (S83F), sul1	gentamicin, streptomycin, ciprofloxacin I/R, nalidixic acid, sulfisoxazole	streptomycin	ColpVC, IncI1
Se_V_46	Chicken	Elche	2016	Kentucky	Kentucky	Kentucky	198	9	36	74	aac(3)-Id, aadA7, gyrA (D87N), gyrA (S83F), sul1	gentamicin, streptomycin, ciprofloxacin I/R, nalidixic acid, sulfisoxazole	streptomycin	ColpVC, IncI1
Se_V_1	Pork	Benaguacil	2016	Derby	Derby	Derby	40	10	37	75	tet(C)	tetracycline	streptomycin	ColE10
Se_V_32	Pork	Valencia	2016	Derby	Derby	Derby	40	10	37	75	tet(C)	tetracycline	streptomycin	ColE10
Se_V_14	Chicken	Algemesí	2017	Typhimurium	Typhimurium	Typhimurium	34	11	38	76	tet(B)	tetracycline	streptomycin, tetracycline,	None
Se_V_16	Chicken	Algemesí	2017	Typhimurium	Typhimurium	Typhimurium	34	11	38	76	tet(B)	tetracycline	streptomycin, tetracycline,	None
Se_V_135	Chicken	Castellón	2017	Chester	Chester	Chester	1954	12	39	77	aph(3'')-Ib, aph(6)-Id, dfrA14, qnrB19, sul2, tet(A)	streptomycin, kanamycin, trimethoprim, ciprofloxacin I/R, sulfisoxazole, tetracycline	streptomycin, tetracycline,	None
Se_V_138	Poultry	Almazora	2017	Chester	Chester	Chester	1954	12	39	77	aph(3'')-Ib, aph(6)-Id, dfrA14, qnrB19, sul2, tet(A)	streptomycin, kanamycin, trimethoprim, ciprofloxacin I/R, sulfisoxazole, tetracycline	streptomycin, tetracycline,	None
Se_V_42	Pork	Vilajoyosa	2016	Typhimurium	Typhimurium	Typhimurium	34	13	41	79	aph(3'')-Ib, aph(6)-Id, blaTEM-1B, sul2, tet(B)	streptomycin, kanamycin, ampicillin, sulfisoxazole, tetracycline	ampicillin streptomycin, tetracycline,	IncQ1
Se_V_43	Pork	Vilajoyosa	2016	Typhimurium	Typhimurium	Typhimurium	34	13	41	79	aph(3'')-Ib, aph(6)-Id, blaTEM-1B, sul2, tet(B)	streptomycin, kanamycin, ampicillin, sulfisoxazole, tetracycline	streptomycin, tetracycline,	IncQ1
Se_V_109	Chicken	Torrent	2017	Bredeney	Bredeney	Bredeney	306	14	42	80	None	Sensitive		None
Se_V_110	Chicken	Torrent	2017	Bredeney	Bredeney	Bredeney	306	14	42	80	None	Sensitive		None
Se_V_114	Turkey	Valencia	2017	Bredeney	Bredeney	Bredeney	306	14	42	80	aadA1, aadA2, cmlA1, sul3, tet(A)	streptomycin, chloramphenicol, sulfisoxazole, tetracycline	streptomycin, tetracycline,	IncI1
Se_V_108	Poultry	Alzira	2010	Bredeney	Bredeney	Bredeney	306	14	56	100	tet(A)	tetracycline	streptomycin, tetracycline,	None
Se_V_100	Chicken	Sueca	2016	Kentucky	Kentucky	Kentucky	198	15	43	81	aac(3)-Id, aadA7, blaTEM-1B, gyrA (D87N), gyrA (S83F), sul1	gentamicin, streptomycin, ampicillin, ciprofloxacin I/R, nalidixic acid, sulfisoxazole	streptomycin	None
Se_V_97	Chicken	Almazora	2016	Kentucky	Kentucky	Kentucky	198	15	43	81	gyrA (D87N), gyrA (S83F)	ciprofloxacin I/R, nalidixic acid	streptomycin	None
Se_V_85	Salad	Valencia	2014	Enteritidis	Enteritidis	Enteritidis	11	16	44	82	gyrA (D87Y)	ciprofloxacin I/R, nalidixic acid		IncFIB(S), IncFII(S)

Se_V_86	Chicken	Valencia	2014	Enteritidis	Enteritidis	Enteritidis	11	16	44	82	gyrA (D87Y)	ciprofloxacin I/R, nalidixic acid		IncFIB(S), IncFII(S)
Se_V_75	Chicken	Valencia	2013	Virchow	Virchow	Virchow	16	17	45	83	gyrA (S83F), qnrD1	ciprofloxacin I/R, nalidixic acid		Col3M
Se_V_76	Chicken	Alzira	2013	Virchow	Virchow	Virchow	16	17	45	83	gyrA (S83F), qnrD1	ciprofloxacin I/R, nalidixic acid	streptomycin	Col3M
Se_V_54	Chicken	Borriana	2017	Infantis	Infantis	Infantis	32	18	46	84	ant(3'')-Ia, gyrA (S83Y), sul1, tet(A)	streptomycin, ciprofloxacin I/R, nalidixic acid, sulfisoxazole, tetracycline	streptomycin, tetracycline,	None
Se_V_55	Chicken	Borriana	2017	Infantis	Infantis	Infantis	32	18	46	84	ant(3'')-Ia, gyrA (S83Y), sul1, tet(A)	streptomycin, ciprofloxacin I/R, nalidixic acid, sulfisoxazole, tetracycline	streptomycin, tetracycline,	None
Se_V_4	Pork	Valencia	2016	Bovismorbificans	Bovismorbificans	Bovismorbificans	142	19	47	85	aph(3'')-Ib, aph(6)-Id, qnrB19, sul2, tet(A)	streptomycin, kanamycin, ciprofloxacin I/R, sulfisoxazole, tetracycline	streptomycin	Col156
Se_V_5	Pork	Valencia	2016	Bovismorbificans	Bovismorbificans	Bovismorbificans	142	19	47	85	aph(3'')-Ib, aph(6)-Id, qnrB19, sul2, tet(A)	streptomycin, kanamycin, ciprofloxacin I/R, sulfisoxazole, tetracycline	streptomycin	Col156
Se_V_133	Chicken	Almazora	2016	Chester	Chester	Chester	1954	20	48	87	aph(3'')-Ib, aph(6)-Id, dfrA14, qnrB19, sul2, tet(A)	streptomycin, kanamycin, trimethoprim, ciprofloxacin I/R, sulfisoxazole, tetracycline	streptomycin, tetracycline,	None
Se_V_134	Poultry	Almazora	2017	Chester	Chester	Chester	1954	20	48	87	aph(3'')-Ib, aph(6)-Id, dfrA14, qnrB19, sul2, tet(A)	streptomycin, kanamycin, trimethoprim, ciprofloxacin I/R, sulfisoxazole, tetracycline	streptomycin, tetracycline,	None
Se_V_137	Poultry	Almazora	2017	Chester	Chester	Chester	1954	20	48	87	aph(3'')-Ib, aph(6)-Id, dfrA14, qnrB19, sul2, tet(A)	streptomycin, kanamycin, trimethoprim, ciprofloxacin I/R, sulfisoxazole, tetracycline	streptomycin, tetracycline,	None
Se_V_89	Pork	Gandia	2017	Enteritidis	Enteritidis	Enteritidis	11	21	50	89	None	Sensitive		IncFIB(S), IncFII(S)
Se_V_87	Oysters	Valencia	2017	Enteritidis	Enteritidis	Enteritidis	11	21	50	90	None	Sensitive		IncFIB(S), IncFII(S)
Se_V_99	Chicken	Valencia	2016	Kentucky	Kentucky	Kentucky	198	22	51	91	aac(3)-Id, aadA7, gyrA (D87N), gyrA (S83F), sul1, tet(A)	gentamicin, streptomycin, ciprofloxacin I/R, nalidixic acid, sulfisoxazole, tetracycline	streptomycin, tetracycline,	None
Se_V_12	Chicken	Crevillent	2016	Kentucky	Kentucky	Kentucky	198	22	51	92	gyrA (D87N), gyrA (S83F)	ciprofloxacin I/R, nalidixic acid	streptomycin	IncI1
Se_V_92	Chicken	Cox	2017	Enteritidis	Enteritidis	Enteritidis	11	23	52	93	None	Sensitive		IncFIB(S), IncFII(S)
Se_V_91	Chicken	Cox	2017	Enteritidis	Enteritidis	Enteritidis	11	23	52	94	None	Sensitive		IncFIB(S), IncFII(S)
Se_V_68	Pork	Valencia	2017	Typhimurium	Typhimurium (PMV)	Typhimurium (PMV)	34	24	53	97	aph(3'')-Ib, aph(6)-Id, blaTEM-1B, sul2	streptomycin, kanamycin, ampicillin, sulfisoxazole	streptomycin	IncQ1
Se_V_67	Pork	Valencia	2017	Typhimurium	Typhimurium (PMV)	Typhimurium (PMV)	34	24	54	98	aph(3'')-Ib, aph(6)-Id, blaTEM-1B, sul2, tet(B)	streptomycin, kanamycin, ampicillin, sulfisoxazole, tetracycline	ampicillin streptomycin, tetracycline,	IncQ1
Se_V_47	Mussel	Valencia	2015	Typhimurium (PMV)	Typhimurium (PMV)	Typhimurium (PMV)	34	24	62	106	aph(3'')-Ib, aph(6)-Id, blaTEM-1B, sul2, tet(B)	streptomycin, kanamycin, ampicillin, sulfisoxazole, tetracycline	ampicillin streptomycin, tetracycline,	IncQ1
Se_V_23	Pork	Pinoso	2016	Derby	Derby	Derby	40	25	59	103	aadA2, dfrA12, sul3, tet(A)	streptomycin, trimethoprim, sulfisoxazole, tetracycline	streptomycin, tetracycline,	p0111
Se_V_22	Pork	Pinoso	2016	Derby	Derby	Derby	40	25	60	104	aadA2, dfrA12, sul3, tet(A)	streptomycin, trimethoprim, sulfisoxazole, tetracycline	streptomycin, tetracycline,	p0111
Se_V_139	Poultry	Valencia	2014	Senftenberg	Llandoff	Jedburgh|Llandoff	5689	26	63	107	qnrB19	ciprofloxacin I/R	streptomycin	None
Se_V_142	Poultry	Valencia	2017	NA	Llandoff	Jedburgh|Llandoff	5689	26	64	108	qnrB19	ciprofloxacin I/R	streptomycin	Col3M

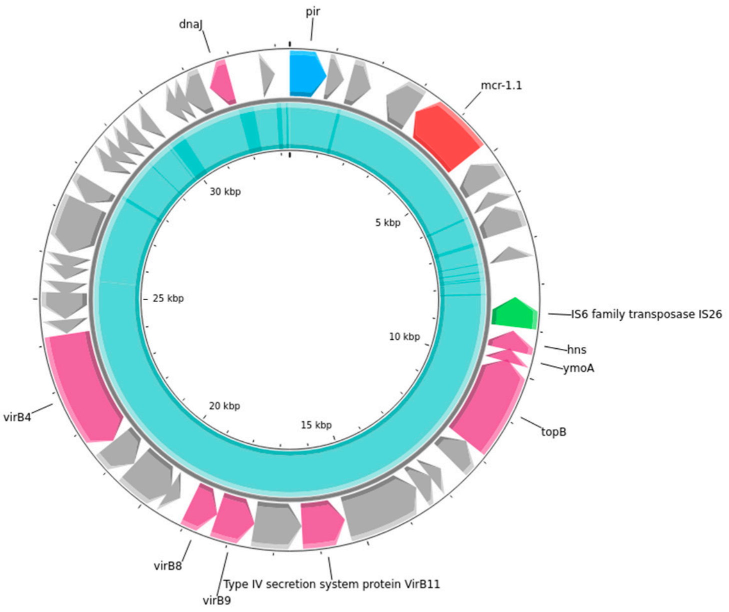

Figure S1. Sequence and structure of the contig containing the *mcr-1* gene with the Incx4 plasmid after comparison with *pGMI17-001_2* of *Salmonella enterica strain CFSAN064033 (NZ_CP028174.1)*.
